# Whole-genome sequencing of *Brassica oleracea* var. *capitata* reveals new diversity of the mitogenome

**DOI:** 10.1371/journal.pone.0194356

**Published:** 2018-03-16

**Authors:** Kiwoung Yang, Ujjal Kumar Nath, Manosh Kumar Biswas, Md Abdul Kayum, Go-eun Yi, Jonghoon Lee, Tae-Jin Yang, Ill-Sup Nou

**Affiliations:** 1 Department of Horticulture, Sunchon National University, Suncheon, Korea; 2 Department of Genetics and Plant Breeding, Bangladesh Agricultural University, Mymensingh, Bangladesh; 3 Joeun Seed, Goesan-Gun, Chungcheongbuk-Do, Republic of Korea; 4 Department of Plant Science, Plant Genomics and Breeding Institute, Research Institute of Agriculture and Life Sciences, College of Agriculture and Life Sciences, Seoul National University, Seoul, Republic of Korea; Chungnam National University, REPUBLIC OF KOREA

## Abstract

Plant mitochondrial genomes (mtDNAs) vary in sequence structure. We assembled the *Brassica oleracea* var. *capitata* mtDNA using a mean coverage depth of 25X whole genome sequencing (WGS) and confirmed the presence of eight contigs/fragments by BLASTZ using the previously reported KJ820683 and AP012988 mtDNA as reference. Assembly of the mtDNA sequence reads resulted in a circular structure of 219,975 bp. Our assembled mtDNA, NCBI acc. no. KU831325, contained 34 protein-coding genes, 3 rRNA genes, and 19 tRNA genes with similarity to the KJ820683 and AP012988 reference mtDNA. No large repeats were found in the KU831325 assembly. However, KU831325 showed differences in the arrangement of bases at different regions compared to the previously reported mtDNAs. In the reference mtDNAs KJ820683 and AP012988, contig/fragment number 4 is partitioned into two contigs/fragments, 4a and 4b. However, contig/fragment number 4 was a single contig/fragment with 29,661 bp in KU831325. PCR and qRT-PCR using flanking markers from separate parts of contig/fragment number 4 confirmed it to be a single contig/fragment. In addition, genome re-alignment of the plastid genome and mtDNAs supported the presence of heteroplasmy and reverse arrangement of the heteroplasmic blocks within the other mtDNAs compared to KU831325 that might be one of the causal factors for its diversity. Our results thus confirm the existence of different mtDNAs in diverse *B*. *oleracea* subspecies.

## Introduction

Several features of plant mitochondrial genomes (mtDNAs) that distinguish them from animal and fungal mtDNAs, include larger genome size, frequent rearrangements, and an extremely low rate of point mutations [1–3]. Plant mtDNAs are relatively stable but complex, and variable in size compared to animal mtDNAs [4–5], their DNA evolves rapidly in structure, but slowly in sequence [6]. The mtDNAs of flowering plants are particularly variable among species in terms of size, structure, and content [7–8]. The angiosperm mtDNAs are typically mapped as circular molecules with one or more large (>1 kb) repeated sequences that promote active homologous recombination [9]. Sequence studies of several plant mitochondrial genes suggest a much lower rate of point mutations than in animal genomes (reviewed by [10–12]). However, the angiosperm mtDNAs are diverse in rates of sequence and structural evolution. Very low synonymous substitution rates are observed in the majority of angiosperm mtDNAs compared to plastid and nucleus [13–15]. Most of the angiosperm mtDNAs are rich in repeat sequences, with moderate to high frequency recombination in the larger repeats [9, 16]. This recombination creates multiple genomic arrangements of varying stoichiometry [8, 17–18] and erodes synteny even among closely related plants within the same species [4, 19–20]. Angiosperm mtDNAs are also enriched with foreign DNAs, might be integrated from plastid and nuclear DNA through intracellular or horizontal transfer [21–22], make them complex.

Despite this, the complexity of mtDNAs in higher plants might be due to the frequent recombination of large and short repeats, resulting in a phenomenon called heteroplasmy that has led to the coexistence of divergent mitochondrial genotypes within a species [17, 23]. In addition, heteroplasmy has co-existence of distinct mitochondrial genomes, but not necessarily due to multipartite structure of this genome, the whole information can be represented in a master chromosome. In plant mitochondrial genome multi-partite configuration has identified as a result of high frequency recombination from repeats [24]. Diversity among mtDNAs may also be due to the presence of sublimons, which are generated by recombinations of short repeats (50–1,000 bp) present in the genome. Quantities of mitochondrial genome sequences and sublimons are varied depending on tissue and organ type and age.

It is important to determine whether a population is introduced or native to a region could be difficult due to inadequate taxonomic characterization, the presence of mysterious lineages, and poor documentation. However, breeders are giving continuous emphasis to enrich the genepool through hybridization by using the diverse germplasm in terms of cytoplasmic or nuclear genomic content of many crop species either introduced or native [25–26]. In *Brassica oleracea* subspecies, most of the cultivars are commercially available as hybrid, of which are produced by using CMS (Cytoplasmic Male Sterile) system [27–28]. *Brassica* species are a rich source of different types of CMS, including ogu CMS [29], pol CMS [30], nap CMS [31], nig CMS [32], and hau CMS [33], which are widely used. Most of them are inherent by mtDNA, therefore, to identify the source materials used in the brassica hybrid breeding programme is important in terms of authenticity and getting expected heterosis. Study on mtDNA diversity would provide valuable information relevant to the introduction of new cytoplasmic genetic variation into target brassica cultivars for its genetic improvement.

The genus *Brassica* includes six important cultivated species whose nuclear genomic relationships are known as the U-triangle [34]. The complete mitochondrial genome sequences of all six species have been reconstructed first time by Chen et al. [35] and found the sequence of *Brassica oleracea* mtDNA with three large repeats (141.8, 3.6, and 2.4 kb) and a total size of approximately 360 kb. More recently, Tanaka et al. [36] and Grewe et al. [37] separately reported two different new *B*. *oleracea* mtDNAs that are shorter than the mtDNA reported by Chang et al. [35]. With the comparison between two mtDNAs, AP012988 or KJ820683 [36–37] and JF920286 [38] suggested that both of the mtDNAs are coexisted in *B*. *oleracea*. Among them, the AP012988 mtDNA is predominant, and the JF920286 mtDNA coexists at a low frequency in all *B*. *oleracea* cultivars are examined [36]. This coexistence of the mtDNA in newly discovered mtDNAs, their domain and motif distribution within the *Brassica* species bring interest to search for more diversity of the mtDNA. Therefore, the aim of this study was to assemble the *B*. *oleracea* var. *capitata* mitochondrial DNA from whole genome sequencing (WGS) and to investigate the genetic diversity compared to the previously published *B*. *oleracea* mtDNAs.

## Materials and methods

### Plant material and genomic DNA extraction

Leaf samples were harvested from four-week-old plants of *B*. *oleracea* var. *capitata* inbred line ‘4119’ grown at Asia Seed Company, Korea. Total genomic DNA was extracted from samples using the DNeasy Plant Mini kit (Qiagen, Germany) following the manufacturer’s protocol. We reconstructed the complete mitochondrial sequence as described below.

### Preparation of whole-genome NGS reads

Whole genome sequencing (WGS) reads of *B*. *oleracea* var. *capitata* were acquired via next-generation sequencing (NGS) using the Illumina HiSeq platform. A paired-end (PE) library with 500-bp inserts was constructed using the Illumina PE DNA library kit according to the manufacturer’s instructions and was sequenced using an Illumina Hiseq2000 sequencing system by the National Instrumentation Center and Environmental Management (NICEM, http://nicem.snu.ac.kr/, Korea) and Macrogen (http://dna.macrogen.com/, Korea). The *B*. *oleracea* genome was completed by using a previously published reference sequence (GenBank accession no. NC_016118) of *B*. *oleracea* genome. The assemblies of WGS reads represented more than 20X genome coverage was considered the cabbage genome as previously described by Kim et al. [39] in rice. From there, we identified contigs/fragments unique to mitochondria using reference mtDNA sequences from NCBI, Acc. Nos. KJ820683, AP012988, and JF920286 of *Brassica*. Mitochondrial contigs/fragments were detected by BLASTN searches [40] for each assembly. The best draft assembly for each species was chosen as the assembly that maximized the total length of mitochondrial contigs/fragments and average length per mitochondrial contig/fragments. The contigs/fragments from our draft assemblies were manually aligned to the reference sequence using BioEdit v7.2.3 [41].

### WGS assembly and building of complete mtDNA sequences

**Fig 1:**
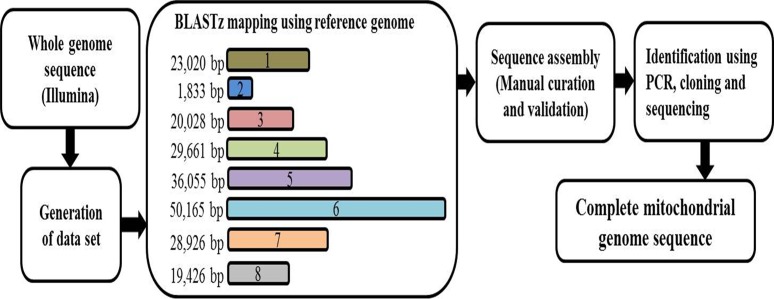
The process for assembling the complete mitochondrial genome sequence using the eight mitochondrial contigs/fragments.

Raw reads with Phred scores of 20 or lower were removed from the total NGS PE reads using the CLC-quality trim tool (quality_trim software included in CLC ASSEMBLY CELL package ver. 4.06 beta. 67189). Sub-data sets were extracted from the trimmed WGS reads and were assembled using CLC *de novo* assembler in the CLC ASSEMBLY CELL package. Sequence gaps were filled by Gap closer with SOAP (Short Oligonucleotide Analysis Package; ver. 1.12). Contigs/fragments representative of the mtDNAs were retrieved from the total contigs/fragments using Nucmer (NUCleotide MUMmer; [42]) with the reference sequences. The retrieved contigs/fragments were ordered and arranged with the related mtDNA sequences based on built-in BLASTZ analysis (http://nature.snu.ac.kr/tools/blastz_v3.php; [43]; Fig 1) and connected into a single draft sequence by joining overlapping terminal sequences. Tentative error sites were identified by mapping raw reads to draft sequences using the CLC mapping tool (clc_ref_assemble in the CLC ASSEMBLY CELL package) and visualized using CLC viewer (clc_assembly_viewer in the CLC ASSEMBLY CELL package). Errors found in repeat, insertion/deletion (InDel), and SNP regions were manually corrected and validated by PCR amplification.

### Annotation and comparative analysis of the mtDNA

The *B*. *oleracea* var. *capitata* mtDNA sequence was annotated using the DOGMA program (http://dogma.ccbb.utexas.edu/) [44] and BLAST searches. A circular map was generated using OGDRAW (http://ogdraw.mpimpgolm.mpg.de/; [45]). The gene structure was reconstructed by comparison with reported sequences and BLAST searches. The syntenic relationship among different mtDNAs of *B*. *olearacea* (NCBI, Acc. Nos. KJ820683, AP012988, JF920286, and KU831325) were constructed by pair-wise alignment with ClustalW and the relationship was visualized using Circos software (http://circos.ca/) [46]. A phylogenic tree was constructed with MEGA6.0 software (http://www.megasoftware.net) [47–48] using the Likelihood method. The evolutionary history was inferred by using the Maximum Likelihood method based on the Tamura-Nei model [49]. Initial tree(s) for the heuristic search were obtained automatically by applying Neighbor-Join and BioNJ algorithms to a matrix of pairwise distances estimated using the Maximum Composite Likelihood (MCL) approach, and then selecting the topology with superior log likelihood value. The analysis involved 5 nucleotide sequences. All positions containing gaps and missing data were eliminated. There were a total of 150244 positions in the final dataset. Evolutionary analyses were conducted in MEGA6 [48].

### Primer design, cloning, and sequencing of the mitocgenome

Differences among the mtDNA contigs/fragments sequences were validated by PCR, cloning, and sequencing using DNA extracted from different sub-genomes of *B*. *oleracea* including commercial cultivars of cauliflower (star 4405), broccoli (SK3-085), cabbage (inbred line 4119, winstorm, CT 623, and CMS based hybrid deabakna), brussels sprouts (cryptus), kohlrabi (siloga), and kailan (blue star). Primers were designed using the web-based Primer3 platform [50]. The PCR conditions included an initial denaturation at 94°C for 5 min followed by 34 cycles of denaturation at 94°C for 30 s, annealing at 58°C for 30 s, and extension at 72°C for 1 min, followed by a final extension at 72°C for 7 min. PCR products were separated by gel electrophoresis on 1.2% agarose gels. The amplified DNA was cloned into the pGEM-T Easy vector (Promega, WI, USA) according to the manufacturer's protocol. The cloned DNA fragments were sequenced by Macrogen Inc. (Seoul, South Korea; www.macrogen.com).

### Expression profiling of different contigs/fragments of the mitocgenome

Total RNA (tRNA) was isolated from five biological replicates of the leaf samples of 4-week old plant using an RNeasy mini kit (Qiagen, Hilden, Germany) and purified with a Qiagen RNase free DNase1 kit. RNA concentrations were measured using NanoDrop^®^ 1000 Spectrophotometer (Wilmington, DE, USA). First-strand cDNA was synthesized using 6 ng tRNA/sample with a Superscript^®^ III First-Strand cDNA synthesis kit (Invitrogen, Carlsbad, CA, USA). Contig/fragment-specific primers for the candidate *B*. *oleracea* mtDNA (Table 1) were designed using Primer3 software (http://frodo.wi.mit.edu/primer3/input.htm). The primers for *Bol-Actin* (F: AAGCCCAAGCAGAGATCAAA, R: CATAACGCCACTCAAGCTCA) from *B*. *oleracea* were used as an internal control. The reaction mixture for qRT-PCR (10 μL total volume) contained 75–80 ng/μL of cDNA, 2 μL forward and reverse primers, 2 μL double distilled water, and 5 μL iTaq^TM^ from the SYBR^®^ Green PCR kit (California, USA). Three replications were used for each qRT-PCR mix. A Light cycler^®^ 96SW 1.1 (Roche, Germany) was used for amplification and detection using the following PCR parameters: pre-denaturation at 95°C for 5 min followed by 40 cycles of 94°C for 10 s, annealing at 58°C for 10 s, and extension at 72°C for 15 s.

**Table 1:** Primers used for identification of the *B*. *oleracea* var. *capitata* mtDNAs.

Primer name	Primer sequence	Product size(bp)	Product identification
Contig 2–6	F: CCCTCTTGAGTAATGAAGAAG	1,458	
	R: ACTGCCCAAGGAAAGAAAAAG		
Contig 5–3	F: CTATCATTAGCTCGGGTAGT	275	KU831325
	R: TTTCGAGTGTGATCAGAAACC		
Contig 4a-4b	F: TCAAAGATCCCACGAACCCA	353	
	R: ACCTCTCCTTTGCTGTTCGA		
Contig 4a-5	F: TCAAAGATCCCACGAACCCA	394	KJ820683,
	R: CCCCGAAAATGCCCGTTAAT		AP012988,
Contig 6-4b	F: CCGTATCAAAGATATTACCACGG	228	JF920286
	R: AGTTCGACTATTGTTACTCC		
Contig 2–3	F: ACAGGCTTGGATACGATCTA	296	
	R: ACCTGCTACGGAACTACCGA		
*Bol-Actin*	F: AAGCCCAAGCAGAGATCAAA	157	Housekeeping gene
	R: CATAACGCCACTCAAGCTCA		

### Sequence alignment for detecting heteroplasmy in mitogenome

*In silico* analysis was performed for detecting heteroplasmy within the different mtDNAs of *B*. *olearacea* (NCBI, Acc. nos. KU831325, KJ820683, JF920286, and AP012988) due to recombination from plastid; and also the block-wise similarities among the mtDNAs. Plastid genome sequence (Acc. no. KR233156) was collected from NCBI (https://www.ncbi.nlm.nih.gov) and used as reference sequence and aligned with the mtDNAs (KU831325, KJ820683, JF920286, and AP012988). Mauve (version 2.4.0) and Geneious Free trial version (https://www.geneious.com/free-trial/) were used to align the genomes and to identify the homology between sequence blocks among the plastid and mtDNA sequences (representated by differential colors); this facilitated to identify the heteroplasmy derived from plastid and simillar regions among the mtDNAs (KU831325, KJ820683, JF920286, and AP012988).

### Data analysis

The 2^−ΔΔCt^ method was used for qRT-PCR data analysis [51]. Relative expression levels of different contigs/fragments were normalized against the expression of the housekeeping gene *Bol-Actin*. Variance analysis and the Tukey test were carried out using SPSS Statistics 17.0 software for Windows to determine the differences of expression of the contigs among various inbred lines of *B*. *oleracea*.

## Results

### Isolation of mtDNA sequences from WGS

**Fig 2:**
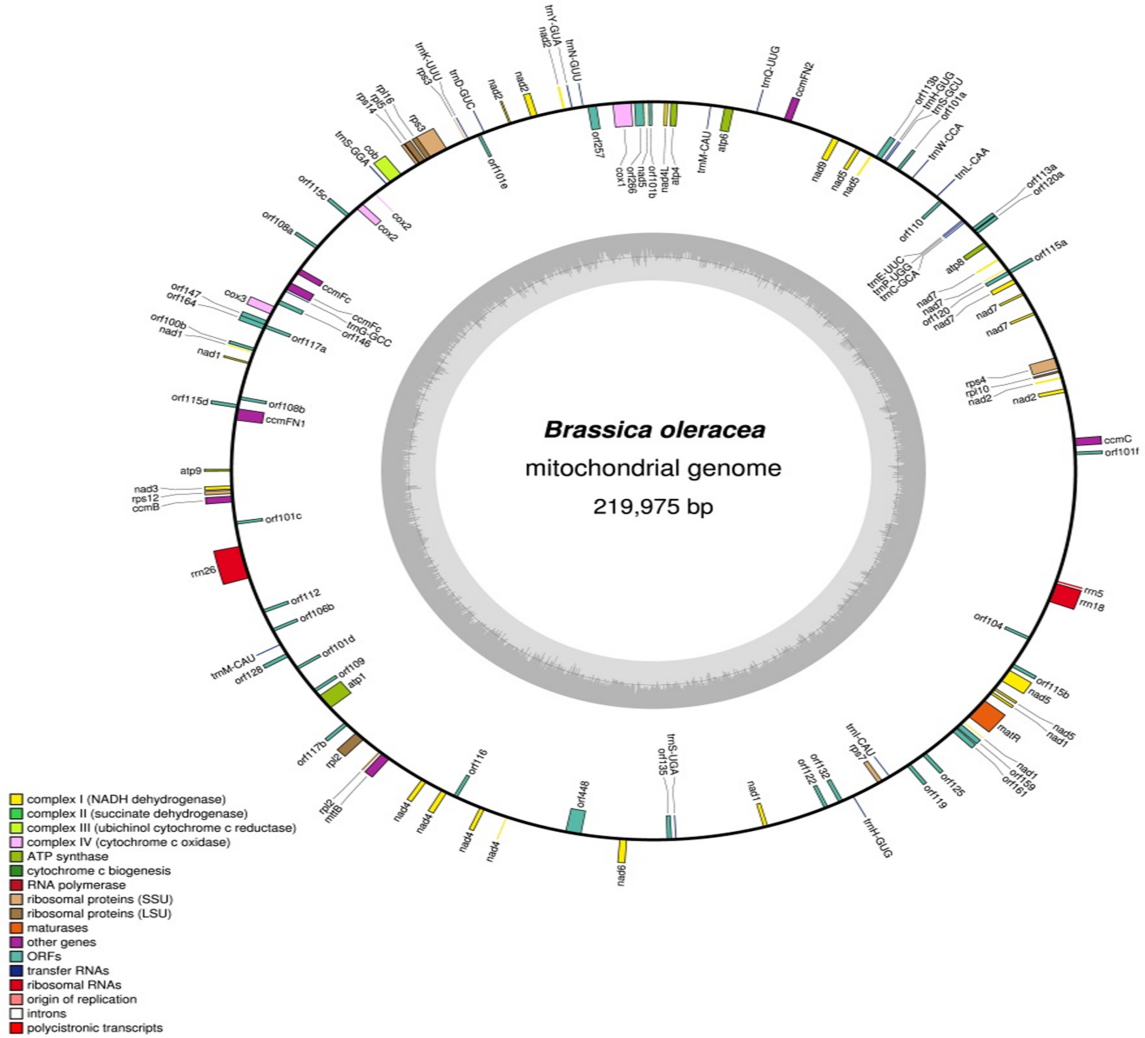
Map of the mitochondrial genome of *B*. *oleracea* var. *capitata*. Boxes on the inside and outside of the outer circle represent ORF genes. Gene colors correspond to the functional categories listed in the legend. The inner circle displays the GC content represented by dark gray bars. The Fig was created using OGDraw v1.2 (adopted from [45]).

**Fig 3:**
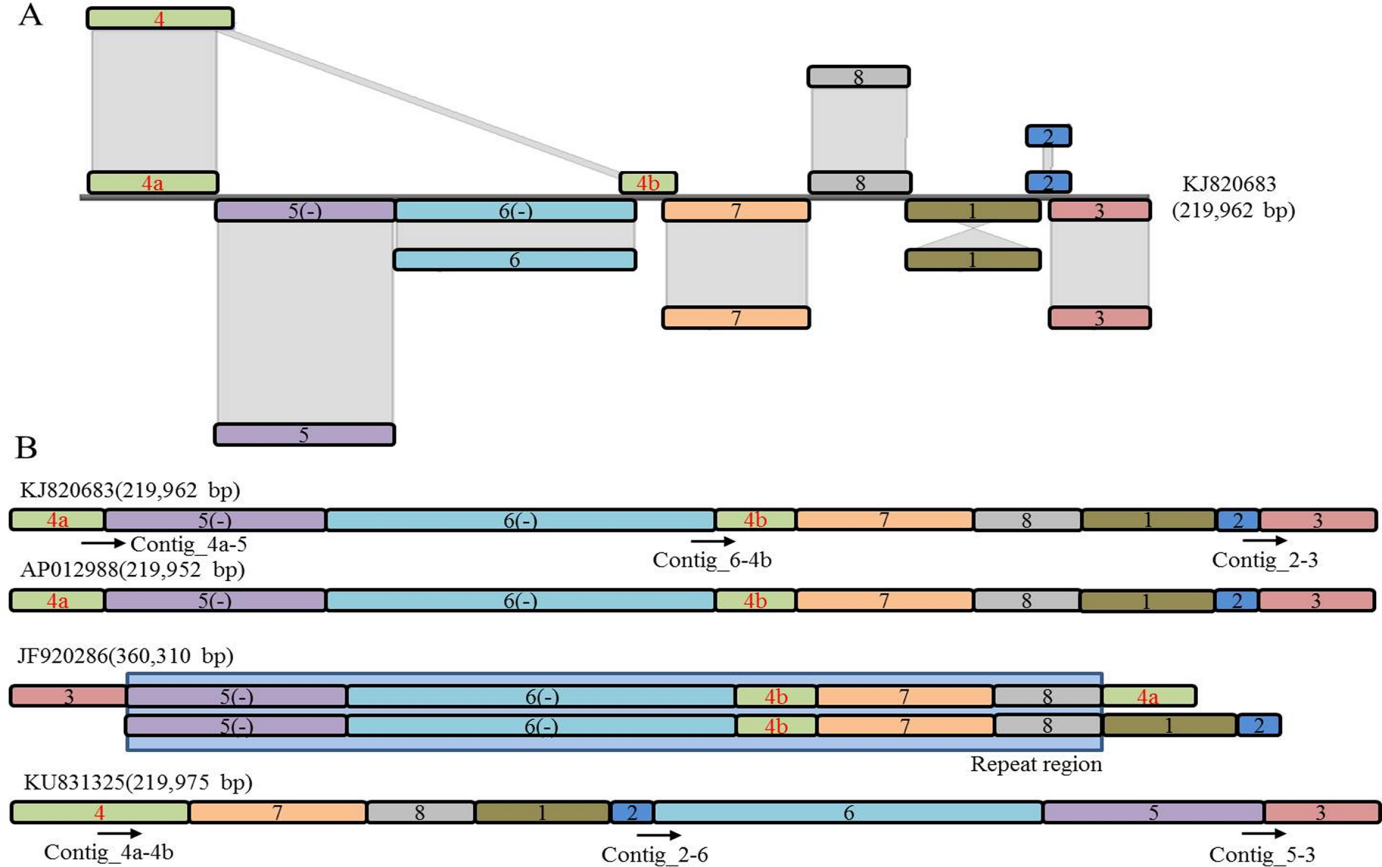
Comparison of KU831325 with the reference mtDNAs KJ820683, AP012988, and JF920286 using the eight related contigs/fragments. (A) The complete mtDNA assembly of *B*. *oleracea* var. *capitata* using the contig/fragment BLASTZ program compared with reference genome KJ820683. Conserved contigs/fragments between KJ820683 and KU831325 are presented with shadow columns (B) Organization of separate regions in the new mtDNA (KU831325) and reference (KJ820683, AP012988, and JF920286) mtDNAs. Minus (-) sign in parenthesis represent the reverse strand. Arrows indicate the designed primer position for distinguishing the mitochondrial contigs/fragments in different genotypes.

We assembled the mtDNA of *B*. *oleracea* var. *capitata* using a mean coverage depth of 20X whole genome sequencing (WGS) (NCBI acc. no. KU831325) and confirmed the presence of ‘eight mitochondrial contigs/fragments’ in *B*. *oleracea* var. *capitata* by BLASTZ using the previously reported ‘KJ820683’ and ‘AP012988’ mtDNAs of *B*. *oleracea* var. *capitata* as reference [39] (Figs 1 and 2). We assembled high-throughput sequencing data of the mtDNA of *B*. *oleracea* var. *capitata* by *de novo* assembly. The whole mtDNA sequence was total 219,975 bp and was assembled into a circular molecule that included ‘eight contigs/fragments’. Among them, contigs/fragments number 6 was the largest (49,020 bp) and contigs/fragments number 2 was the smallest (1,833 bp) (Figs 1 and 2). Interestingly, we have found deviated distribution of the mitochondrial contigs/fragments in our newly submitted NCBI acc. no. KU831325 with reverse order of the contigs/fragments 4, 8, and 2 compared to the previously published reference mtDNAs of *B*. *oleracea* (Fig 3A). Our WGS and assembly showed contig/fragment number 1–2 connected to contig/fragment number 2–6, and contig/fragment number 2–6 connected to contig/fragment number 5–3 (Fig 3B), which differed from the reference mitochondrial genomes KJ820683, AP012988, and JF920286. Furthermore, in the reference genomes KJ820683 and AP012988, contig/fragment number 4 was partitioned into two separate sub-contigs/fragments (4a and 4b). However, contig/fragment number 4 was appeared as single contig/fragment with 29,661 bp in our reported KU831325 mtDNA (Fig 3B). This genome contained 34 protein-coding genes, 3 rRNA genes, and 19 tRNA genes, which is as similar to the reference mtDNAs KJ820683 and AP012988 (S1 Table). We also detected 43 open reading frames (ORFs) in our *B*. *oleracea* mtDNA. The sequencing coverage was approximately 25X (S1 Fig). The overall GC content was 45.25%, which was also alike to the reference mtDNAs KJ820683 and AP012988.

### Comparison of the new mtDNA with the reference mtDNA

**Fig 4:**
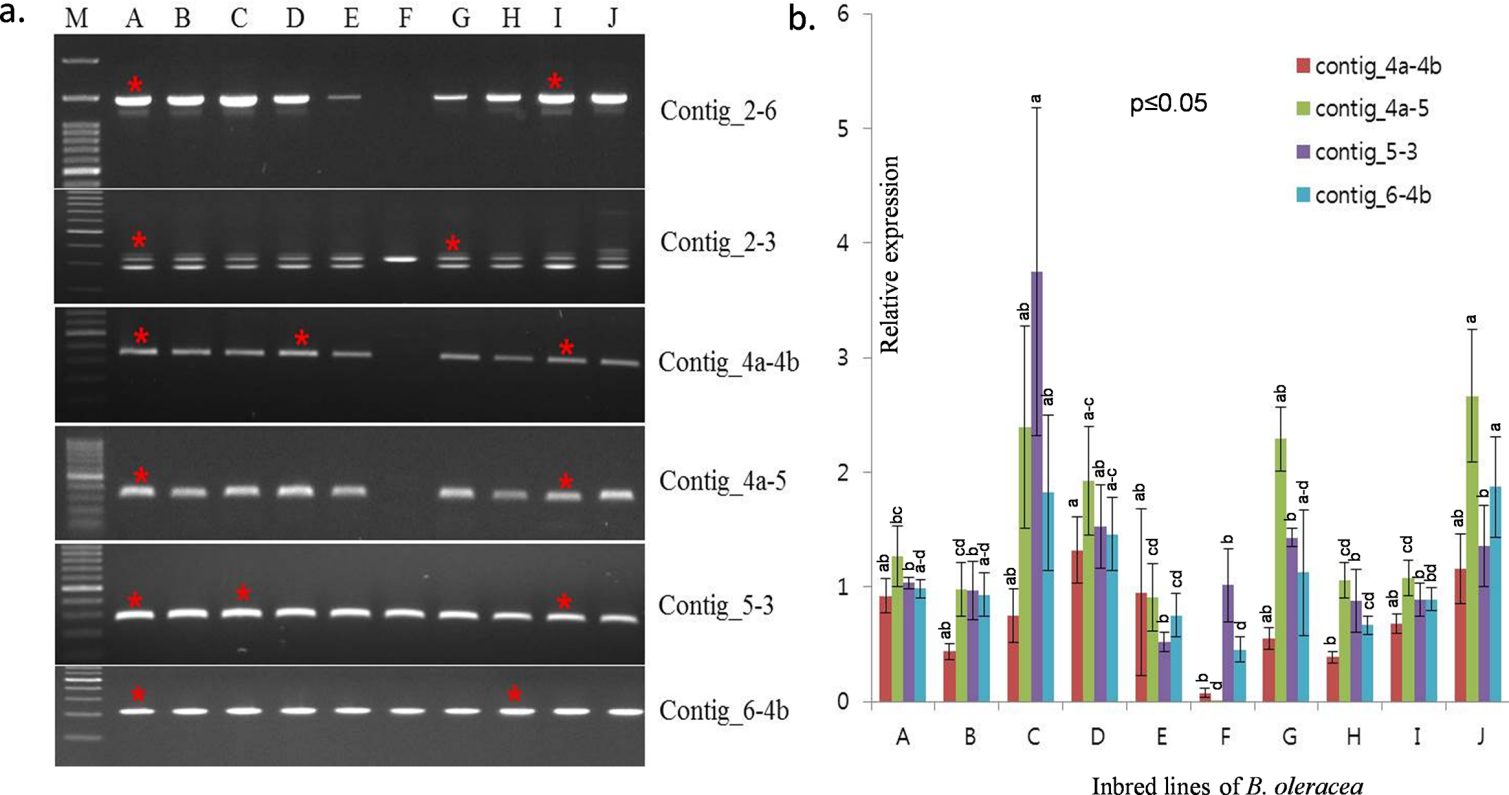
In the left panel (a) identification of variation in the KU831325 mtDNA in various inbred lines. A, B, and E are cabbage, C is broccoli, D is kale, F is cabbage of the Ogura CMS line, G is cauliflower, H is brussels sprouts, I is kohlrabi, and J is kailan and M is the reference ladder with 100 bp. The genotypes with the red asterisks were used for cloning, sequencing, and assemblage of mitochondrial DNA in *B*. *oleracea*. There are two bands for contig 2–3, with the lower band being the target amplified according to the primer product size presented in Table 1. In right panel (b) showing relative expression with significant variation of different contigs/fragments present in different genotypes.

The size of the predicted genome suggested the presence of same contigs/fragments and restriction map of *Brassica* species as in the reference mtDNAs. However, while the mtDNAs reported by Tanaka et al. [36] and Grewe et al. [37] shared a common sequence and structural alignment, while the mtDNA of *B*. *oleracea* reported in here showed structural variations in the contigs/fragments (Fig 3A and 3B). Primers designed at the flanking regions of contigs/fragments 2–6, 5–3, and 4a-4b were used to confirm the presence of the contigs/fragments in different individuals covering subspecies of *B*. *oleracea* (Fig 4A and 4B). In our reported mtDNA contig/fragment number 4 (4a-4b) was found in all of the tested *Brassica* genotypes except Ogura male sterile cabbage line (Fig 4A). We also looked for whether both of the previously reported mtDNAs of AP012988 or KJ820683 are present in our reported *B*. *oleracea* var. *capitata* mtDNA or not. The amplification of the flanking primer of contig/fragment 4a-5 confirmed the coexistence of both of the previously reported mtDNAs (Fig 4A and 4B). The differential rearrangement of contigs/fragments might be due to the presence of large and short repeat sequences within the mtDNAs of the tested *B*. *oleracea* var. *capitata* lines. To extent the results of coexistence of the previously reported mtDNAs, a BLAST search was carried out for contigs/fragments 2–6, 5–3, and 4a-4b, which also confirmed their coexistence with higher similarity (100%) with three previously published *B*. *oleracea* mtDNA. The other contigs/fragments also exhibited similarity (99%) with previously published *B*. *oleracea* mtDNAs (S2 Fig).

### Expression pattern of contigs/fragments of the new mtDNA compared to other mtDNAs

The four contigs/fragments, 4a-4b, 4a-5, 5–3, and 6-4b showed differential expression in all ten tested *B*. *oleracea* genotypes. The expression profile of 4a-4b was almost absent in the Ogura male-sterile cabbage line compared to other tested *B*. *oleracea* materials (Fig 4B). The expression of contigs/fragments 4a-5 and 6-4b were significantly (*p≤0*.*05*) higher than contigs/fragments 4a-4b in cabbage, broccoli, the Ogura male-sterile cabbage line, and brussels sprouts. However, the highest expression was found in contig/fragment 5–3 in broccoli. The expression of contig/fragment 4a-5 was also significantly (*p≤0*.*05*) different from contig/fragment 4a-4b in cabbage, broccoli, the Ogura male-sterile cabbage line, cauliflower, and brussels sprouts (Fig 4B). There was no significant (*p≤0*.*05*) difference in relative expression among contigs/fragments 4a-4b, 4a-5, 5–3, and 6-4b for cabbage and kale. The relative expression of contig/fragment 4a-5 was also significantly (*p≤0*.*05*) higher than contig/fragment 4a-4b in cabbage, broccoli, cauliflower, brussels sprouts, kohlrabi, and kailan. No expression of contig/fragment 4a-5 was found in the Ogura type male-sterile cabbage line (Fig 4B).

### Heteroplasmy from plastid into mtDNAs

**Fig 5:**
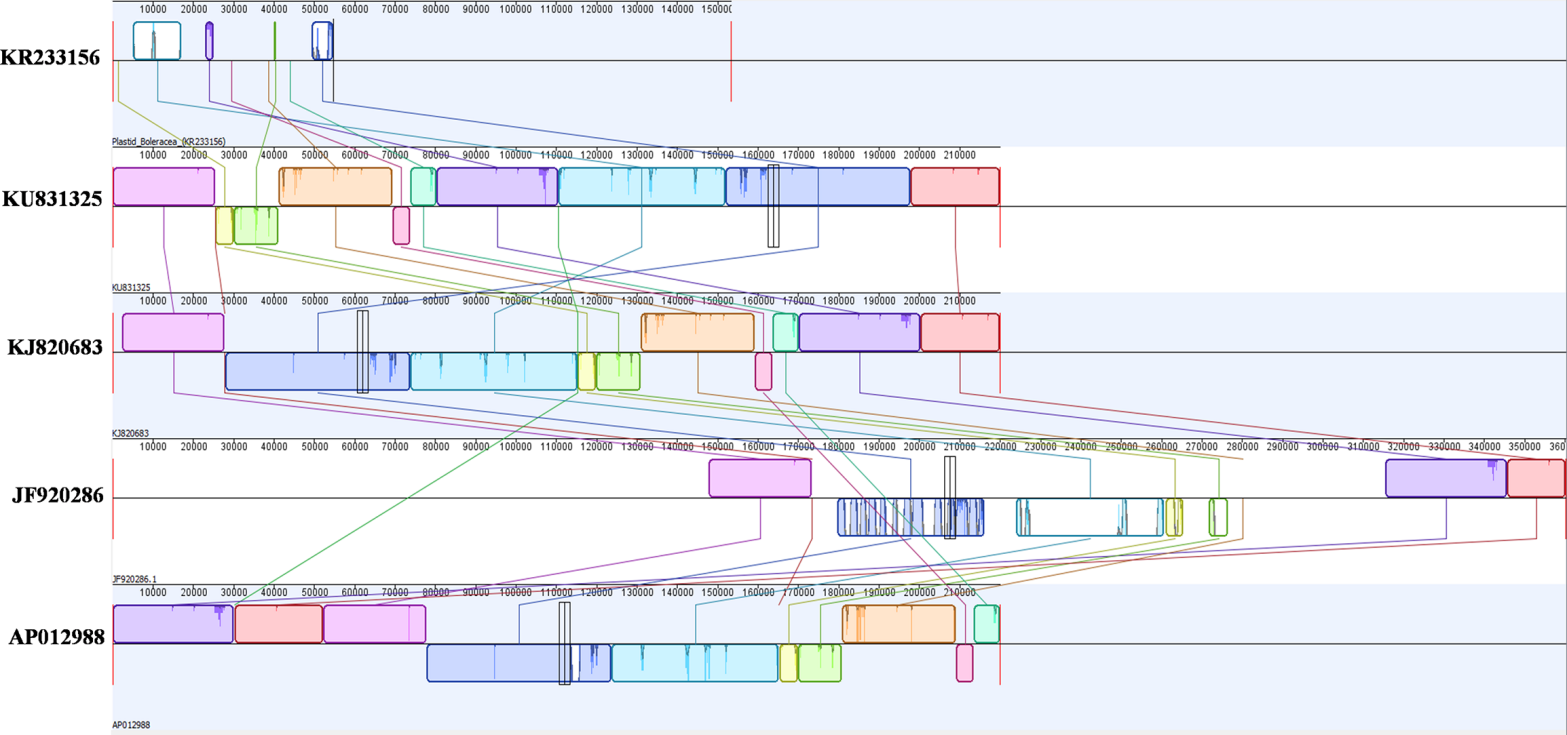
A multiple alignment of plastid genome (KR233156) and four mtDNAs (KU831325, KJ820683, JF920286, and AP012988) of *Brassica oleracea* consists of several rearranged pieces. Each genome is laid out horizontally with homologous blocks represented as coloured rectangles. Regions inverted relative to plastid are set below those that match in the forward orientation. Lines collate aligned segments between plastid and different mtDNAs. Sections of white within blocks and gaps between blocks indicate lineage specific sequence.

Whole-genome re-alignments of mtDNAs (KU831325, KJ820683, JF920286, and AP012988) with plastid genome sequence (Acc. no. KR233156) was performed to find out the heteroplasmy in the mtDNAs from plastid due to recombinations, and also for the similarities among the mtDNAs with our reported mtDNA KU831325. We have identified 12 blocks in the mtDNAs KU831325, KJ820683, and AP012988, whereas only nine blocks were deployed in mtDNA JF920286, when aligned with the plastid genome sequence (KR233156) (Fig 5 and Table 2). The similar blocks in the different mtDNAs are represented by the same colour in the Fig 5. However, the direction and position of different blocks in the genome were completely different between KU831325 Vs KJ820683 and KU831325 Vs AP012988. Among the 12 blocks, five blocks were arranged as reverse order in the mtDNAs KJ820683 and AP012988, whereas, they were in normal direction in the mtDNA KU831325. In addition, herteroplasmy from plastid genome was not detected for block 1, block 3, block 11, and block 12 in the mtDNAs KU831325, KJ820683, and AP012988 except mtDNA JF920286, in where block 3, block 6, and block 9 were completely absent (Table 2). Rest of the blocks had wide range of hetroplasmy, the highest heteroplasmy was detected in the block 5 and block 10 with 2182 bp and 1547 bp, respectively in the mtDNA KU831325. Considering the whole plastid genome size, the highest hetroplasmy was also presented in the mtDNA KU831325 in terms of total bases (5519 bp out of 153366 bp of plastid; Table 2) and in terms of percent (3.6%; S2 Table). By contrast, the re-alignment of mtDNAs KJ820683, AP012988, and JF920286 in reference with KU831325 showed more than 98% similarities with KJ820683 and AP012988 (S2 Table). Therefore, it could be concluded that our reported mtDNA KU831325 is a diverse type mtDNA in *B*. *oleracea* might be due to presence of either heteroplasmy in terms of higher recombination of plastid genome or the rearrangement and positions of the blocks within the mtDNA.

**Table 2:** Block-wise amino acid similarities with the plastid (Plst) nucleotide sequences (bp) into mitochondrial (Mt) genome in *B*. *oleracea* and the nucleotide similarities of the KU831325 with other reference mitogenomes (Mts).

References	Mt genome	Block 1	Block 2	Block 3	Block 4	Block 5	Block 6	Block 7	Block 8	Block 9	Block 10	Block 11	Block 12	Total
Plastid genome (KR233156) Vs.	KU831325	0.0	134	0.0	643	2182	45	89	654	225	1547	0.0	0.0	5519
	KJ820683	0.0	134	0.0	643	2147	45	89	648	225	1520	0.0	0.0	5451
	JF920286	0.0	134	-	254	2147	-	0.0	0.0	-	1407	0.0	0.0	3942
	AP012988	0.0	134	0.0	643	2147	45	89	648	225	207	0.0	0.0	4138
Mt genome (KU831325) Vs.	KJ820683	46	4591	56	41446	30129	4473	28199	11014	6405	45920	25454	19694	217427
	JF920286	46	3341	-	4743	30127	-	100	1191	-	21153	25454	14607	100762
	AP012988	26	4620	56	41378	30130	4473	28199	11016	6405	43758	25453	22104	217618

### Structural differences in the mtDNAs

The new *B*. *oleracea* mtDNA contained 34 protein-coding genes, 3 rRNA genes, and 19 tRNA genes (S1 Table) that were presumably inherited from the angiosperm common ancestor. In addition to known genes, angiosperms mtDNAs also contained numerous ORFs of unknown function. Thirty-eight of these ORFs were found as conserved in our reported new *B*. *oleracea* var. *capitata* mtDNA as the member of angiosperm (Fig 2).

### Repeats and syntenic regions in the mtDNAs

**Fig 6:**
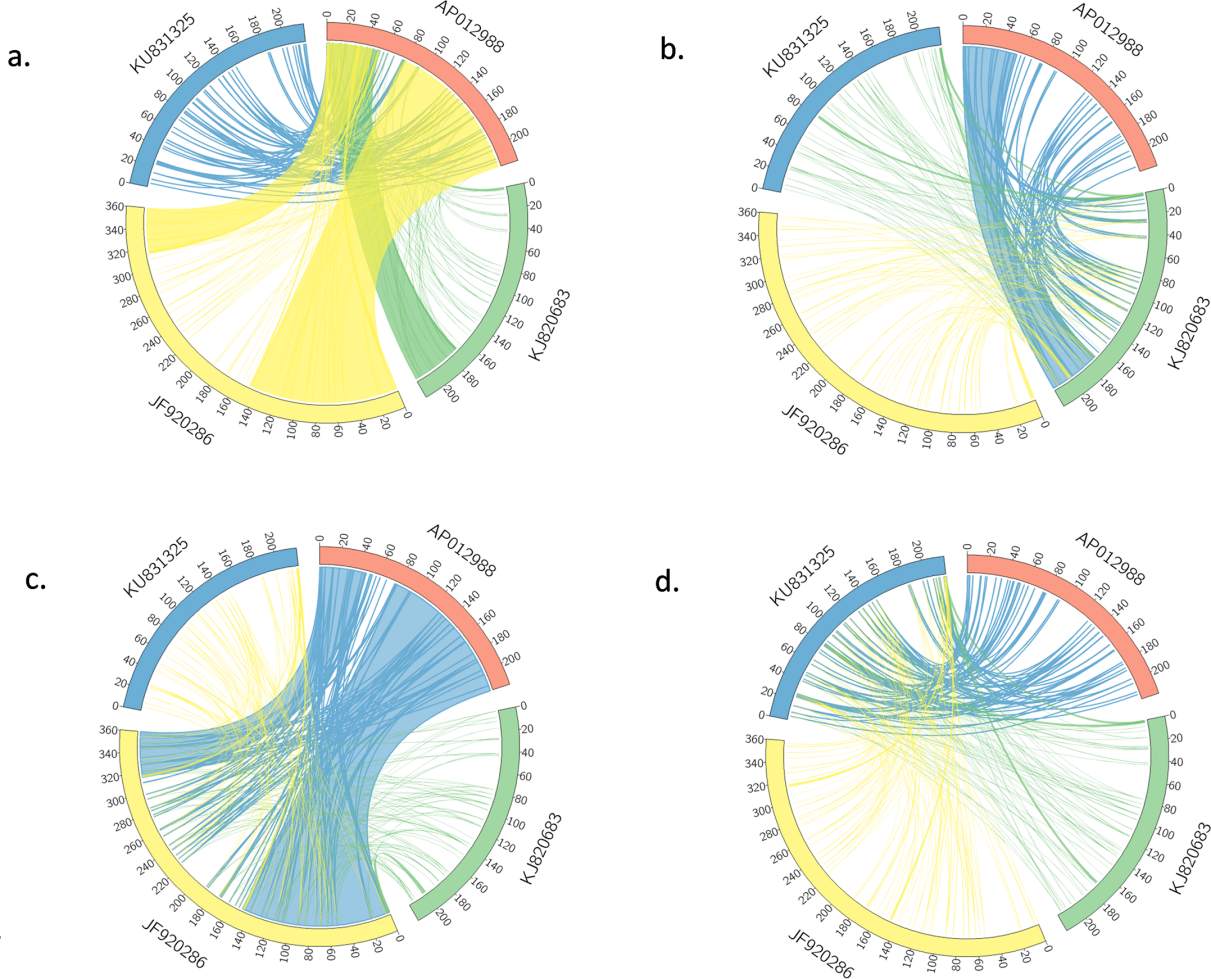
Syntenic block comparative analysis in *Brassica oleracea* mtDNAs. The map was generated using Circos. (a) Syntenic block of *B*. *oleracea* mtDNA ‘AP012988’ with three other *B*. *oleracea* mtDNAs. (b) Syntenic block of *B*. *oleracea* mtDNA ‘KJ820683’ with three other *B*. *oleracea* mtDNAs. (c) Syntenic block of *B*. *oleracea* mtDNA ‘JF920286’ with three other *B*. *oleracea* mtDNAs. (d) Syntenic block of *B*. *oleracea* mtDNA ‘KU831325’ with three other *B*. *oleracea* mtDNAs.

**Fig 7:**
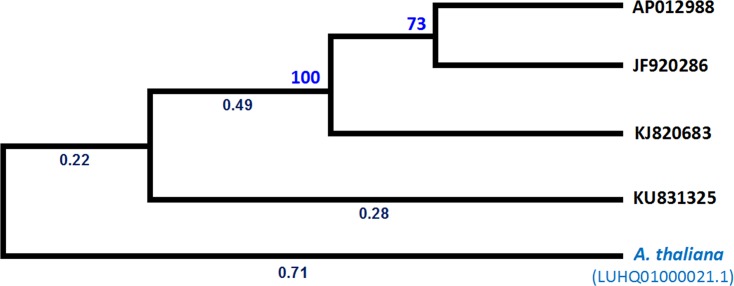
Molecular phylogenetic analysis by Maximum Likelihood method of the four *B*. *oleracea* mtDNAs according to the genomic sequences and the distribution of their contigs, the black coloured value is node length and blue coloured one is boost-strap value.

The complex, multipartite structure of the *Brassica* mitochondrial genome was formed due to presence and recombination of repeats [6]. No large repeats were identified in KU831325 but 101 tandem repeats were confirmed, where the repeat length was 2,178 bp/repeat (S3 Table). Tandem repeats similar to those in KU831325 also exist in the reference mtDNAs KJ820683 and AP012988, but not in the JF920286. Frequency distribution analysis of the mtDNAs showed that the size of the tandem repeats was ranged between 8 and 24 bp (S3 Fig). The short tandem repeats accounted for about 1.1% of the entire *B*. *oleracea* mtDNA. Short repeats are associated with an irreversible organization and uniformly distributed throughout the *Brassica* mtDNAs [35, 52]. To investigate the diversity and access mitochondrial genome structure variation among the four *B*. *oleracea* mtDNAs, microsyntenic analysis was performed. The syntenic sequence blocks were identified as syntenic fragments with variable size in length among the mtDNAs. However, in the mtDNA KU831325 had about 2.4 kb syntenic blocks when compared with other three mtDNAs (S4 Table). The previously reported *B*. *oleracea* mtDNAs, AP012988, JF920286, and KJ820683 had good linearity to each other. Whereas, our reported mtDNA KU831325 showed little amount of linearity with AP012988, JF920286, and KJ820683 (Fig 6). A total of 77, 117, and 74 syntenic blocks were identified in case of KU831325 compared with AP012988, JF920286, and KJ820683, respectively (S4 Table). All the syntenic comparisons in *Brassica* mitochondrial genomes are shown in the Circos map (Fig 6A–6D). A large duplicated region identified in JF920286 was absent from KU831325, AP012988, and KJ820683. We found that some rearrangements in block regions might be associated with short repeats. Phylogenetic analysis of the four *B*. *oleracea* mtDNAs with *Arabidopsis thaliana* (LUHQ01000021.1) as out-group showed that AP012988, JF920286, and KJ820683 were distributed within one group, but KU831325 was in a separate class (Fig 7). This diversity indicated that KU831325 is likely a new mtDNA, not previously described.

## Discussion

Mitochondria contribute to energy production; thus, metabolism and cell homeostasis depend on the performance of the mitochondrial genetic system [53]. Plant mitochondrial DNA comprises a set of sub-genomic forms called sublimons with a mixture of linear, circular, and branched structures [54]. To elucidate the mitochondrial genomic diversity within a species of *Brassica*, we sequenced the mtDNA of *B*. *oleracea* var. *capitata*. Diversity in mtDNAs might be due to evolutionary factors such as duplications, rearrangements, InDels, and mutations [3, 19, 55–56]. In this study, we identified a different type of *B*. *oleracea* mtDNA sequence with a different distribution of contigs/fragments compared to the reference mtDNAs (KJ820683, AP012988, and JF920286) of *B*. *oleracea*. Our reported mtDNA (KU831325) is slightly larger than KJ820683 and AP012988 based on previously reported data from physical mapping [6, 36–37] of *B*. *oleracea* (Fig 2). However, this mtDNA is much smaller than the previously reported ‘JF820683’ mtDNA. The ‘JF920286’-type mtDNA (360,271 bp) reported by Chang et al. [38] is larger than our mtDNA due to the presence of a large repeat (140 kbp). Our reported mtDNA has eight characteristic contigs/fragments like the previously published other *B*. *oleracea* mtDNAs [35–37]. In addition, contig/fragment number 4, which is presumed to be a combination of contigs/fragments 4a and 4b, showed 92 to 100% similarity with the mtDNA of different reported NCBI accessions of different *Brassica* species (S2A Fig). Contigs/fragments 2–6 had 99% similarity with different reported accessions of *B*. *juncea*, *B*. *rapa*, *B*. *napus*, and *B*. *oleracea*, and contig/fragment 5–3 also showed 99 to 100% similarity with reported mtDNAs of different *Brassica* species (S2B and S2C Fig). Most of the ‘KU831325’-type genes may exist in several copies because of many short tandem repeat regions and are identical to those of the other previously reported mtDNAs. The only exception was contig/fragment number 4, which we have found absent in the previously reported KJ820683, AP012988, and JF920286 mtDNAs, might be due to DNA rearrangement in the mtDNA. Recombination is an essential process for shaping and maintaining diversity in the genome, and may be the basis of generation of new diversity in mtDNAs [53]. In our reported mtDNA KU831325, we have found comparatively larger heteroplasmy from plastid DNA and completely different types of distribution and direction of some heteroplasmic blocks compared to other reference mtDNAs (Table 2 and Fig 5) might make the diverse type of mtDNA KU831325.

MtDNAs are typically mapped as circular molecules with one or more large repeated sequences that promote active homologous recombination [9]. In our reported circular mtDNA of *B*. *oleracea* about 99 ORFs we have found, among them 34 ORFs are protein coding gene, 3 rRNA genes, and 19 tRNA genes; rest of the ORFs could not be characterized for their definite functions (Fig 2 and S1 Table). This result indicates, in addition to known genes, *B*. *oleracea* mtDNAs contain numerous ORFs of unknown function. Several of these ORFs are conserved among diverse higher plant species and are functional mitochondrial genes [57]. Some ORFs have been shown to play a role in establishing cytoplasmic male sterility (CMS), although the majority of these CMS-associated ORFs are species specific and are likely to affect male fertility by mechanisms unique to each species [58–59]. Besides ORFs, we have found the presence of large subunit (LSU) and small subunit (SSU) of ribosomal proteins might be arisen through inter- and intra-genomic recombination in plant species. Such inter- and intra-genomic recombination can lead to dynamic structural diversity consisting of multiple, coexisting mitogenomic forms, as evidenced by DNA gel blot mapping, cosmid sequencing, and read-pair mapping [8, 17, 60], though a few species lack large mitochondrial repeats and presumably exist in a single predominant conformation [4, 61–62]. Mitochondrial gene content can also vary among angiosperms [7, 63]. Some species, such as the tulip tree *Liriodendron tulipifera* [64], have retained all 41 protein-coding genes that were presumably present in the common angiosperm ancestor, but most present-day species have retained only a subset of these genes. Mitochondrial gene loss is commonly linked to the ongoing transfer of DNA to the nucleus, which mostly affects genes for ribosomal proteins and the subunits of the succinate dehydrogenase complex [65]. However, most mitochondrial ORFs are not conserved among angiosperms [8, 60, 66–67] and are generally considered to be nonfunctional [63].

The comparison of the *B*. *oleracea* mtDNAs revealed that the KU831325 mtDNA shared 77, 177, and 74 syntenic blocks with AP012988, JF920286, and KJ820683, respectively, where the syntenic average block size was ranged 105–162 bp. By contrast, the syntenic average block size among AP012988, JF920286, and KJ820683 was ranged 134–1943 bp with very large single syntenic block up to 142 kb in AP012988 vs JF920286 (S4 Table). We also identified short tandem repeats of 2,424 bp with a repeat length of 8 to 24 bp (S3 Fig), which may have been involved in the reorganization of the ‘KU831325’ mtDNA. These repeats can also explain the rearrangement between the ‘AP012988’ and ‘KU831325’ mtDNAs via homologous recombination (Fig 6D). Alverson et al. [17] reported that plant mitochondrial genomes are rich in repeated sequences. Mitochondrial repeated sequences could have originated via recombination of the shorter repeats, producing sublimons in plants. Differential expression profiling of the newly identified ‘contig/fragment number 4 (4a-4b)’ showed lower expression than other contigs/fragments, even within the individual member of the *B*. *oleracea* genotypes reveal the presence of a separate contig/fragment in the reported mtDNA of *B*. *oleracea* (Fig 4B).

The ratios between the abundances of sublimons in the KU831325, AP012988, and KJ820683 mtDNAs are varied. Sublimons are generated by recombination of short repeats and are sometimes amplified rapidly, replacing the main genome by a substoichiometric shifting process, while the original genome is suppressed to a low-frequency level [68–71]. Substoichiometric shifting is possibly caused by environmental conditions or mutations in the nuclear recombination proofreading machinery [9, 52, 72].

In conclusion, the KU831325 mtDNA sequence reported here a different variety of mtDNAs in *B*. *oleracea*, thereby, coexistence of the JF920286 mtDNAs is usually at low frequency compared to KU831325 (Table 2). Phylogenetic analysis of *B*. *oleracea* mitochondrial genome suggested that the KU831325 mtDNA is a diverse type mtDNA compared to AP012988 and KJ820683 (Fig 7). The diversity of the present mtDNA also confirmed by its comparable size with already published *B*. *oleracea* mtDNA. In addition, presence and distribution of hetroplasmic and syntenic blocks also support the diversity of the new *B*. *oleracea* mtDNA reported here.

## Supporting information

S1 Fig Mitochondrial whole genome sequence. Average cover depth is approximately 20X.
(TIF)

S2 Fig Identification of the sequence of contig/fragment 4 by NCBI BLASTn and alignment for analysis with the sequences of contig/frgament 4a-4b (A), and other attached contigs/fragments with contig/fragment 4 (B & C) in different *Brassica* species.
(TIF)

S3 Fig Frequency distribution of short repeats in KU831325. (TIF)

S1 Table Gene contents, size, and feature of the *Brassica oleracea* mtDNAs. (DOC)

S2 Table Block-wise percent heteroplasmy due to recombination from the plastid (Plst) nucleotide sequences into mitochondrial (Mt) genome in *B*. *oleracea* and similarities of the KU831325 with other reference mitogenomes (Mts). (DOC)

S3 Table Comparison of the tandem repeat distribution in the mitochondrial genomes of KU831325 and the three reference accessions of *B*. *oleracea*. (DOC)

S4 Table Comparative features of syntenic block distribution and their nucleotide coverage in the total genome among different *B*. *oleracea* mtDNAs. (DOC)
