# Supplementary figures and images for: Whole-genome sequencing of Brassica oleracea var. capitata reveals new diversity of the mitogenome
Source: PLoS One. 2018 Mar 16;13(3):e0194356. doi: 10.1371/journal.pone.0194356 (PMC5856397; doi:10.1371/journal.pone.0194356)

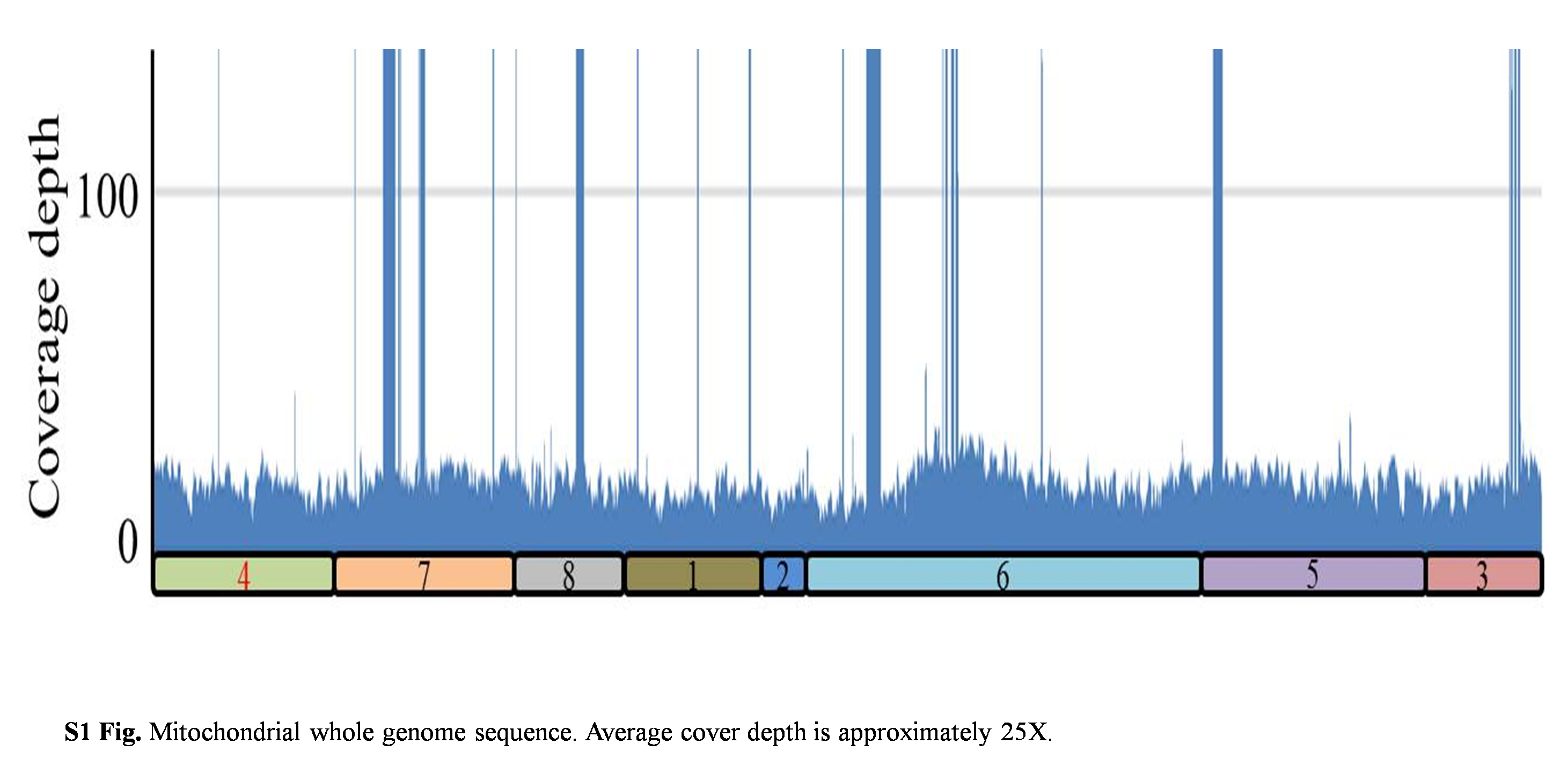

Supplement: S1 Fig — Average cover depth is approximately 20X. (TIF) [file pone.0194356.s001.tif]

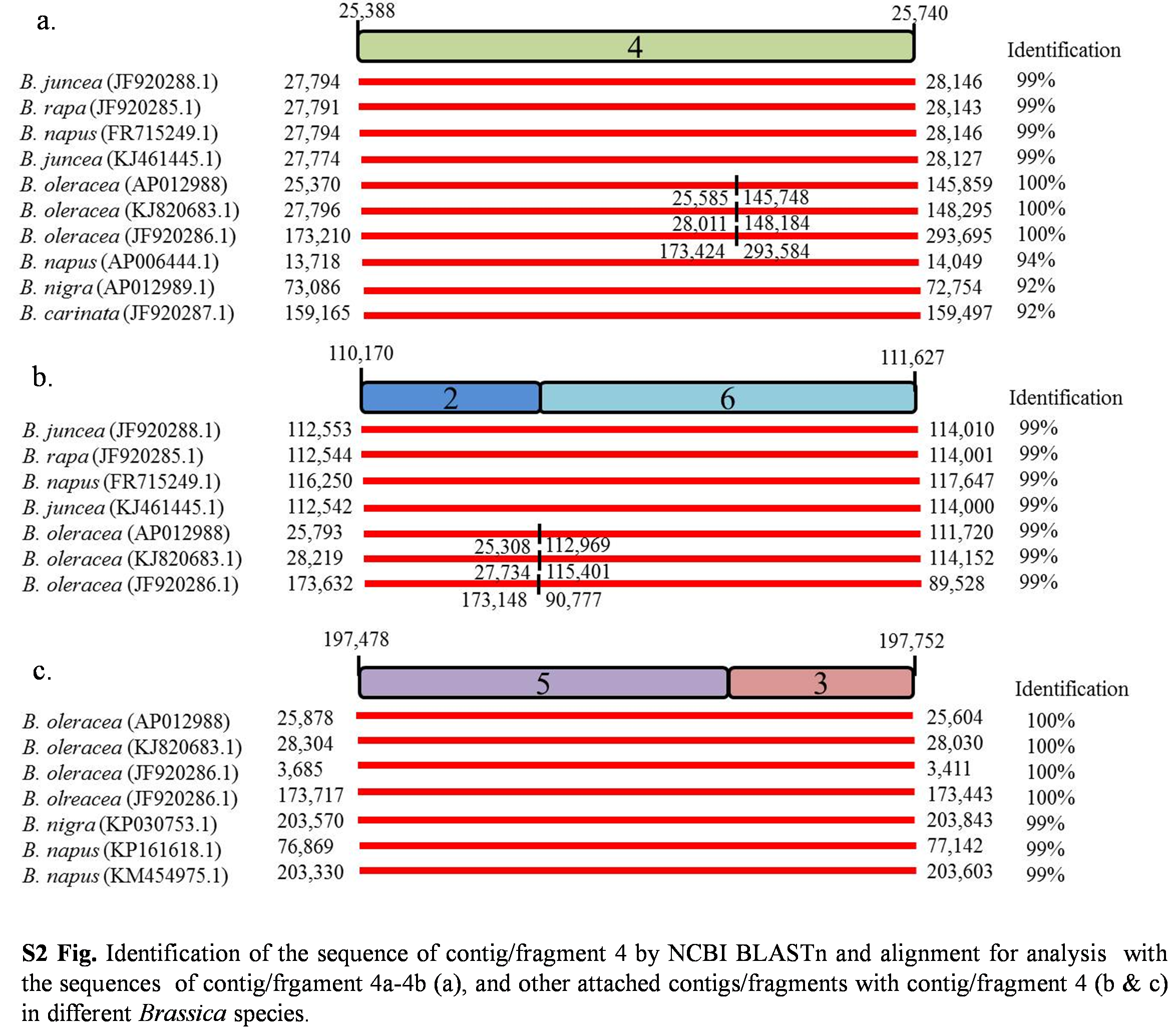

Supplement: S2 Fig — Identification of the sequence of contig/fragment 4 by NCBI BLASTn and alignment for analysis with the sequences of contig/frgament 4a-4b (A), and other attached contigs/fragments with contig/fragment 4 (B & C) in different Brassica species. (TIF) [file pone.0194356.s002.tif]

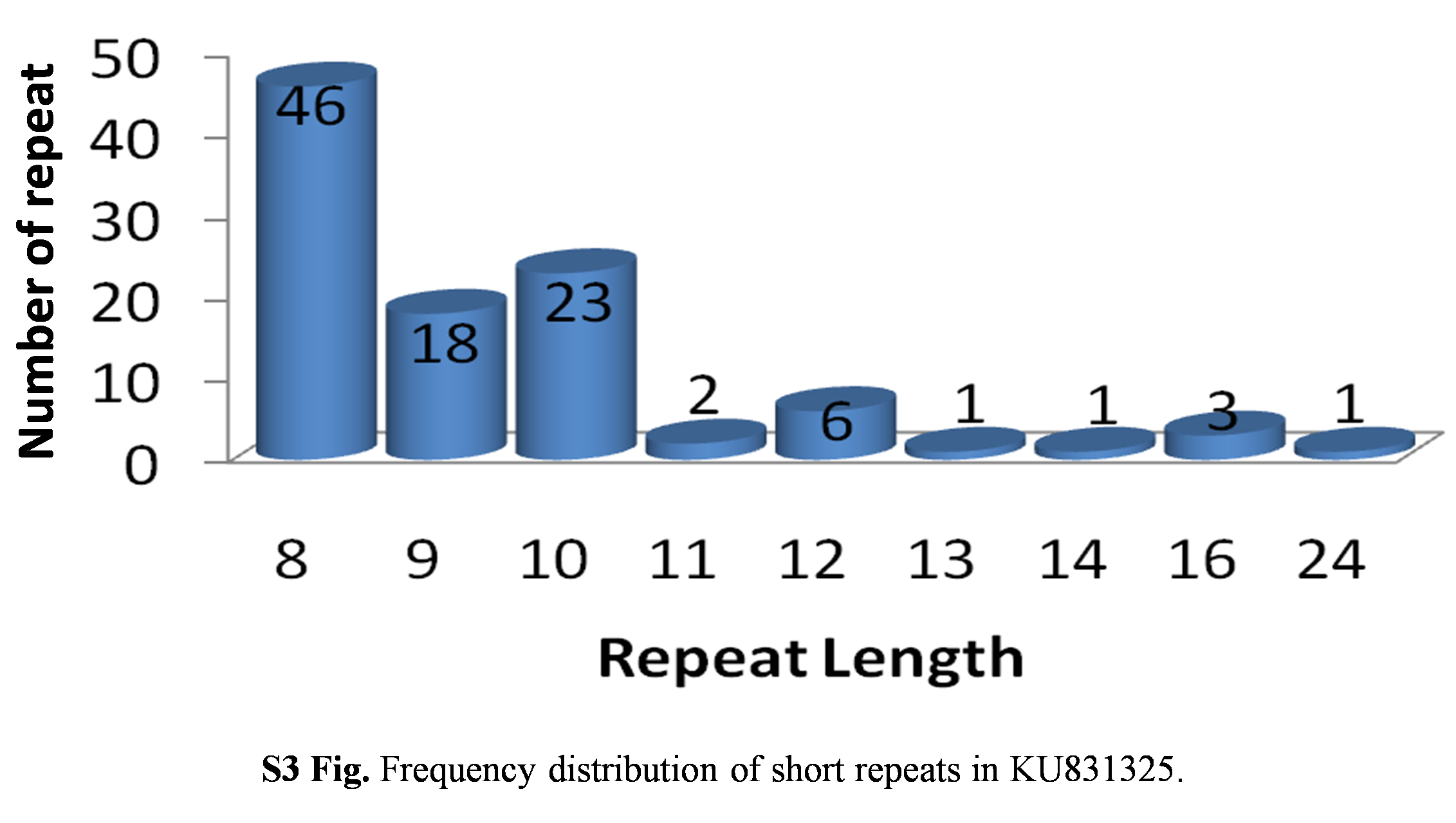

Supplement: S3 Fig — (TIF) [file pone.0194356.s003.tif]
