# Supplementary material for: Whole-genome sequencing of Brassica oleracea var. capitata reveals new diversity of the mitogenome
Source: PLoS One. 2018 Mar 16;13(3):e0194356. doi: 10.1371/journal.pone.0194356 (PMC5856397; doi:10.1371/journal.pone.0194356)
Supplement: S1 Table — (DOC) [file pone.0194356.s004.doc]

**S1 Table**. Gene contents, size, and feature of the *Brassica oleracea* mitogenomes.

| **Functional  category** | **Gene** | **KU831325** | | | **KJ820683** | | | **AP012988** | | |
| --- | --- | --- | --- | --- | --- | --- | --- | --- | --- | --- |
|  |  | **Size (bp)** | **Strand** | **Feature  (Copy number)** | **Size (bp)** | **Str**  **and** | **Feature  (Copy number)** | **Size (bp)** | **Str**  **and** | **Feature  (Copy number)** |
| Complex I | *nad1* | 260 | R | exon5 (1) | 260 | F | exon5 (1) | 260 | F | exon5 (1) |
|  |  | 58 | R | exon4 (1) | 58 | F | exon4 (1) | 58 | F | exon4 (1) |
|  |  | 387 | R | exon1 (1) | 387 | F | exon1 (1) | 388 | F | exon1 (1) |
|  |  | 81 | F | exon2 (1) | 81 | F | exon2 (1) | 80 | F | exon2 (1) |
|  |  | 192 | F | exon3 (1) | 192 | F | exon3 (1) | 192 | F | exon3 (1) |
|  | *nad2* | 392 | F | exon2 (1) | 392 | F | exon2 (1) | 392 | F | exon2 (1) |
|  |  | 153 | F | exon1 (1) | 153 | F | exon1 (1) | 153 | F | exon1 (1) |
|  |  | 161 | F | exon3 (1) | 161 | F | exon3 (1) | 161 | F | exon3 (1) |
|  |  | 573 | F | exon4 (1) | 573 | F | exon4 (1) | 573 | F | exon4 (1) |
|  |  | 188 | F | exon5 (1) | 188 | F | exon5 (1) | 188 | F | exon5 (1) |
|  | *nad3* | 357 | R | (1) | 357 | F | (1) | 357 | F | (1) |
|  | *nad4* | 90 | F | exon4 (1) | 90 | R | exon4 (1) | 90 | R | exon4 (1) |
|  |  | 422 | F | exon3 (1) | 422 | R | exon3 (1) | 422 | R | exon3 (1) |
|  |  | 515 | F | exon2 (1) | 515 | R | exon2 (1) | 515 | R | exon2 (1) |
|  |  | 461 | F | exon1 (1) | 461 | R | exon1 (1) | 461 | R | exon1 (1) |
|  | *nad4L* | 303 | F | (1) | 303 | F | (1) | 303 | F | (1) |
|  | *nad5* | 115 | R | (2) | 115 | F/R | (2) | 115 | F/R | (2) |
|  |  | 28 | R | (2) | 28 | F/R | (2) | 28 | F/R | (2) |
|  |  | 148 | F | exon5 (1) | 148 | F | exon5 (1) | 148 | F | exon5 (1) |
|  |  | 394 | F | exon4 (1) | 394 | F | exon4 (1) | 394 | F | exon4 (1) |
|  |  | 22 | F | exon3 (1) | 22 | F | exon3 (1) | 22 | F | exon3 (1) |
|  |  | 229 | F | exon1 (2) | 229 | F | exon1 (1) | 229 | F | exon1 (1) |
|  |  | 1217 | F | exon2 (1) | 1217 | F | exon2 (1) | 1217 | F | exon2 (1) |
|  | *nad6* | 618 | R | (1) | 618 | F | (1) | 618 | F | (1) |
|  |  | 105 | F | (1) | 105 | F | (1) | 105 | F | (1) |
|  | *nad7* | 262 | R | exon5 (1) | 262 | R | exon5 (1) | 262 | R | exon5 (1) |
|  |  | 244 | R | exon4 (1) | 244 | R | exon4 (1) | 244 | R | exon4 (1) |
|  |  | 467 | R | exon3 (1) | 467 | R | exon3 (1) | 467 | R | exon3 (1) |
|  |  | 69 | R | exon2 (1) | 69 | R | exon2 (1) | 69 | R | exon2 (1) |
|  |  | 143 | R | exon1 (1) | 143 | R | exon1 (1) | 143 | R | exon1 (1) |
|  | *nad9* | 573 | F | (1) | 573 | F | (1) | 573 | F | (1) |
| Complex III | *cob* | 1182 | F | (1) | 1182 | F | (1) | 1182 | F | (1) |
| Complex IV | *cox1* | 1584 | F | (1) | 1584 | F | (1) | 1584 | F | (1) |
|  | *cox2* | 2074 | F | (2) | 2074 | F | (2) | 2074 | F | (2) |
|  |  | 83 | R | exon2 (2) | 83 | R | exon2 (2) | 83 | R | exon2 (2) |
|  |  | 700 | R | exon1 (2) | 700 | R | exon1 (2) | 700 | R | exon1 (2) |
|  |  | 2672 | F | (1) | 2672 | F | (1) | 2672 | R | (1) |
|  | *cox3* | 31 | R | (1) | 31 | F | (1) | 31 | F | (1) |
|  |  | 798 | F | (1) | 798 | F | (1) | 798 | F | (1) |
| Complex V | *atp1* | 1524 | R | (1) | 1524 | F | (1) | 1524 | F | (1) |
|  | *atp4* | 579 | F | (1) | 579 | F | (1) | 579 | F | (1) |
|  | *atp6* | 786 | F | (1) | 786 | F | (1) | 786 | F | (1) |
|  | *atp8* | 477 | F | (1) | 477 | F | (1) | 477 | F | (1) |
|  | *atp9* | 225 | F | (1) | 225 | F | (1) | 225 | F | (1) |
| Cytochrome c | *ccmB* | 621 | R | (1) | 621 | F | (1) | 621 | F | (1) |
|  | *ccmC* | 744 | F | (1) | 744 | F | (1) | 744 | F | (1) |
|  | *ccmFc* | 579 | R | exon2 (1) | 550 | R | exon2 (1) | 549 | R | exon2 (1) |
|  |  | 783 | R | exon1 (1) | 779 | R | exon1 (1) | 780 | R | exon1 (1) |
|  | *ccmFN1* | 1146 | F | (1) | 1146 | F | (1) | 1146 | F | (1) |
|  | *ccmFN2* | 636 | F | (1) | 636 | F | (1) | 636 | F | (1) |
|  |  | 27 | F | (1) | 27 | F | (1) | 27 | F | (1) |
| Ribosome | *rpl2* | 133 | F | exon2 (1) | 133 | R | exon2 (1) | 133 | R | exon2 (1) |
|  |  | 917 | F | exon1 (1) | 917 | R | exon1 (1) | 917 | R | exon1 (1) |
|  | *rpl5* | 558 | F | (1) | 558 | F | (1) | 558 | F | (1) |
|  | *rpl10* | 152 | F | (1) | 152 | F | (1) | 152 | F | (1) |
|  | *rpl16* | 540 | F | (1) | 435 | F | (1) | 540 | F | (1) |
|  | *rps3* | 75 | F | exon1 (1) | 74 | F | exon1 (1) | 75 | F | exon1 (1) |
|  |  | 1590 | F | exon2 (1) | 1591 | F | exon2 (1) | 1590 | F | exon2 (1) |
|  | *rps4* | 1089 | F | (1) | 1089 | F | (1) | 1089 | F | (1) |
|  | *rps7* | 447 | R | (1) | 447 | F | (1) | 447 | F | (1) |
|  | *rps12* | 378 | F | (1) | 378 | F | (1) | 378 | F | (1) |
|  |  | 42 | F | (1) | 42 | F | (2) | 42 | F | (2) |
|  | *rps14* | 303 | F | (1) | 303 | F | (1) | 303 | F | (1) |
| rRNA | *rrn5* | 119 | F | (1) | 119 | F | (1) | 119 | F | (1) |
|  | *rrn18* | 1848 | F | (1) | 1848 | F | (1) | 1848 | F | (1) |
|  | *rrn26* | 3148 | R | (1) | 3148 | F | (1) | 3148 | F | (1) |
| tRNA | *trnC-GCA* | 71 | F | (1) | 71 | F | (1) | 70 | F | (1) |
|  | *trnD-GUC* | 74 | F | (1) | 74 | F | (1) | 74 | F | (1) |
|  | *trnE-UUC* | 72 | F | (1) | 72 | F | (1) | 72 | F | (1) |
|  | *trnfM-CAU* | 74 | R | (1) | 74 | F | (1) | 74 | F | (1) |
|  | *trnG-GCC* | 72 | F | (1) | 72 | F | (1) | 72 | F | (1) |
|  | *trnH-GUG* | 74 | R | (1) | 74 | F | (1) | 74 | F | (1) |
|  | *trnH-GUG* | 74 | F | (1) | 74 | F | (1) | 74 | F | (1) |
|  | *trnI-CAU* | 74 | R | (1) | 81 | F | (1) | 81 | F | (1) |
|  | *trnK-UUU* | 73 | F | (1) | 73 | F | (1) | 73 | F | (1) |
|  | *trnL-CAA* | 85 | F | (1) | 85 | F | (1) | 85 | F | (1) |
|  | *trnM-CAU* | 73 | F | (1) | 73 | F | (1) | 73 | F | (1) |
|  | *trnM-CAU* | 74 | F | (1) | 74 | F | (1) | 74 | F | (1) |
|  | *trnN-GUU* | 72 | F | (1) | 72 | F | (1) | 72 | F | (1) |
|  | *trnP-UGG* | 75 | F | (1) | 75 | F | (1) | 75 | F | (1) |
|  | *trnQ-UUG* | 72 | F | (1) | 72 | F | (1) | 72 | F | (1) |
|  | *trnS-GCU* | 88 | F | (1) | 88 | F | (1) | 88 | F | (1) |
|  | *trnS-GGA* | 87 | F | (1) | 87 | F | (1) | 87 | F | (1) |
|  | *trnS-UGA* | 87 | R | (1) | 87 | F | (1) | 87 | F | (1) |
|  | *trnW-CCA* | 74 | F | (1) | 74 | F | (1) | 74 | F | (1) |
|  | *trnY-GUA* | 83 | F | (1) | 83 | F | (1) | 83 | F | (1) |
| Other | *matR* | 2034 | R | (1) | 1974 | F | (1) | 2034 | F | (1) |
|  | *mttB* | 810 | F | (1) | 810 | F | (1) | 810 | F | (1) |
|  | *sdh4* | 405 | F | (1) | 405 | F | (1) | 405 | F | (1) |
| Total size |  | 42,594 | | | 42,403 | | | 42,567 | | |
