# Supplementary material for: Whole-genome sequencing of Brassica oleracea var. capitata reveals new diversity of the mitogenome
Source: PLoS One. 2018 Mar 16;13(3):e0194356. doi: 10.1371/journal.pone.0194356 (PMC5856397; doi:10.1371/journal.pone.0194356)
Supplement: S2 Table — (DOC) [file pone.0194356.s005.doc]

**S2 Table.** Block-wise percent heteroplasmy due to recombination from the plastid (Plst) nucleotide sequences into mitochondrial (Mt) genome in *B. oleracea* and similarities of the KU831325 with other reference mitogenomes (Mts).

| References | Mt genome | Block 1 | Block 2 | Block 3 | Block 4 | Block 5 | Block 6 | Block 7 | Block 8 | Block 9 | Block 10 | Block 11 | Block 12 | Total from |
| --- | --- | --- | --- | --- | --- | --- | --- | --- | --- | --- | --- | --- | --- | --- |
| Plastid genome (KR233156) Vs. | KU831325 | 0.0 | 46.36 | 0.0 | 1.43 | 99.91 | 97.83 | 96.74 | 95.47 | 54.75 | 15.83 | 0.0 | 0.0 | 3.60 |
|  | KJ820683 | 0.0 | 46.36 | 0.0 | 1.43 | 98.31 | 97.83 | 96.74 | 94.60 | 54.75 | 15.55 | 0.0 | 0.0 | 3.55 |
|  | JF920286 | 0.0 | 46.36 | - | 0.56 | 98.31 | - | 0.0 | 0.0 | - | 14.04 | 0.0 | 0.0 | 2.57 |
|  | AP012988 | 0.0 | 46.36 | 0.0 | 1.43 | 98.31 | 97.83 | 96.74 | 94.60 | 54.75 | 1.82 | 0.0 | 0.0 | 2.70 |
| Mt genome (KU831325) Vs. | KJ820683 | 95.83 | 100 | 100 | 100 | 99.88 | 100 | 100 | 99.83 | 100 | 99.94 | 100 | 89.11 | 98.84 |
|  | JF920286 | 95.83 | 65.42 | - | 6.89 | 99.87 | - | 39.37 | 20.98 | - | 40.62 | 100 | 66.07 | 45.81 |
|  | AP012988 | 100 | 99.98 | 100 | 99.96 | 99.88 | 100 | 99.46 | 99.91 | 100 | 91.02 | 99.99 | 99.99 | 98.92 |
