# Supplementary material for: Whole-genome sequencing of Brassica oleracea var. capitata reveals new diversity of the mitogenome
Source: PLoS One. 2018 Mar 16;13(3):e0194356. doi: 10.1371/journal.pone.0194356 (PMC5856397; doi:10.1371/journal.pone.0194356)
Supplement: S3 Table — (DOC) [file pone.0194356.s006.doc]

**S3 Table.** Comparison of the tandem repeat distribution in the mitochondrial genomes of KU831325 and the three reference accessions of *B. oleracea*.

| Result of tandem repeats search | AP012988 | JF920286 | KJ820683 | KU831325 |
| --- | --- | --- | --- | --- |
| Total size of mito sequences (bp) | 219952 | 360271 | 219962 | 219975 |
| Total number of identified tandem repeats | 101 | 163 | 101 | 101 |
| Repeat density | 2178 | 2210 | 2178 | 2178 |
| Class II Repeat | 100 | 162 | 100 | 100 |
| Class I Repeat | 1 | 1 | 1 | 1 |
| AT rich SSR: | 78 | 125 | 78 | 78 |
| GC rich SSR: | 16 | 25 | 16 | 16 |
| AT/GC balance: | 7 | 13 | 7 | 7 |
| Mono nucleotide repeats | 84 | 137 | 84 | 84 |
| Di nucleotide repeats | 11 | 20 | 11 | 11 |
| Tri nucleotide repeats | 5 | 6 | 5 | 5 |
| Tetra nucleotide repeats | 1 | 0 | 1 | 1 |
| Penta nucleotide repeats | 0 | 0 | 0 | 0 |
| Hexa nucleotide repeats | 0 | 0 | 0 | 0 |
