# Supplementary material for: Whole-genome sequencing of Brassica oleracea var. capitata reveals new diversity of the mitogenome
Source: PLoS One. 2018 Mar 16;13(3):e0194356. doi: 10.1371/journal.pone.0194356 (PMC5856397; doi:10.1371/journal.pone.0194356)
Supplement: S4 Table — (DOC) [file pone.0194356.s007.doc]

**S4 Table**. Comparative features of syntenic block distribution and their nucleotide coverage in the total genome among different *B. oleracea* mitogenomes.

| Mitogenome combination | Number of  Syntenic  block | Syntenic block size  range  (bp) | Average  Syntenic block  size (bp) | Total number of nucleotide in homology (% of coverage) | |
| --- | --- | --- | --- | --- | --- |
| KU831325 Vs AP012988 | 77 | 2429-34 | 126 | at KU = 9722 (4%) | at AP = 9722 (4%) |
| KU831325 Vs JF920286 | 117 | 2407-34 | 105 | at KU = 12343 (6%) | at JF = 12344 (3%) |
| KU831325 Vs KJ820683 | 74 | 2429-34 | 162 | at KU = 11991 (5%) | at KJ = 11991 (5%) |
| AP012988 Vs JF920286 | 104 | 142120-34 | 1943 | at AP = 202153 (92%) | at JF = 202158 (56%) |
| AP012988 Vs KJ820683 | 67 | 49913-34 | 886 | at AP = 59383 (27%) | at KJ = 59382 (27%) |
| JF920286 Vs KJ820683 | 88 | 3600-34 | 134 | at JF = 9734 (3%) | at KJ = 9735 (4%) |
